# Factors shaping cleaning and disinfection practices during the COVID-19 pandemic: A qualitative evidence synthesis

**DOI:** 10.4102/jphia.v16i2.624

**Published:** 2025-02-22

**Authors:** Ogonna N.O. Nwankwo, Anne N. Meremikwu, Ezinne C. Okebe, Marcel A. Otonkue, Hope N. Okebalama, Kathleen Dunn, Hannah Hamilton-Hurwitz, April Baller

**Affiliations:** 1 Department of Community Medicine, Faculty of Clinical Sciences, University of Calabar, Calabar, Nigeria; 2 Department of Public Health and Community Medicine, University of Calabar Teaching Hospital, Calabar, Nigeria; 3 Swiss Centre for International Health, Swiss Tropical and Public Health Institute, Allschwil, Switzerland; 4 University of Basel, Basel, Switzerland; 5 Department of Science and Vocational Education, Faculty of Education, University of Calabar, Calabar, Nigeria; 6 Cochrane Nigeria, Institute of Tropical Diseases Research and Prevention, University of Calabar Teaching Hospital, Calabar, Nigeria; 7 World Health Organization, Geneva, Switzerland; 8 Public Health Agency of Canada, Ottawa, Canada

**Keywords:** COVID-19, qualitative evidence synthesis, systematic review, rapid appraisal, infection prevention and control, cleaning, disinfection

## Abstract

**Background:**

Cleaning and disinfection of the physical environment is important as it can reduce the transmission of microorganisms. However, adherence to cleaning and disinfection protocols varies due to factors such as personal factors and external influences like resource availability, workload, and institutional support.

**Aim:**

To synthesise factors influencing the uptake of cleaning and disinfection interventions in healthcare and community setting in the context of COVID-19.

**Setting:**

These findings as seen in any country irrespective of setting.

**Method:**

Medline and World Health Organization (WHO) COVID-19 Research databases were searched from January 2020 to September 2022. The search identified 1618 studies, and analysis was performed using the thematic synthesis approach. The confidence in each review finding was ascertained using the Grading of Recommendations, Assessment, Development, and Evaluations-Confidence in the Evidence from Reviews of Qualitative Research (GRADE-CERQual) approach.

**Results:**

Six analytical themes were identified. Cleaning and disinfection were seen as a cornerstone of patient care. Individual judgement, historic standards, norms and practices, ability to implement rapid practice guideline change and resource considerations were seen to influence the uptake of cleaning.

**Conclusion:**

There is a need for further qualitative studies in these areas, especially looking at the different interventions from an equity lens. Resource needs and availability were key factors influencing the uptake of cleaning and disinfection in both communities and health facilities.

**Contribution:**

This review shows important considerations for implementing infection prevention and control (IPC) interventions in the context of COVID-19.

## Introduction

Adherence to infection prevention and control (IPC) strategies, which encompass, early recognition, source control, administrative controls, environmental/engineering controls and personal protective equipment (PPE), is imperative to limiting the spread of severe acute respiratory syndrome coronavirus 2 (SARS-CoV-2) within health facilities.^1^ In addition, public health and social measures, like physical distancing, mask use, and cleaning and disinfection are critical to reducing community spread of SARS-CoV-2. Cleaning and disinfecting the physical environment is vital for reducing the spread of microorganisms. The immediate environment of an infected individual may serve as a source of transmission, as infectious SARS-CoV-2 has been found on environmental surfaces; making proper cleaning and disinfection critical.^2,3^ Coronavirus disease 2019 (COVID-19) is vulnerable to surfactants in cleaning agents and disinfectants.^4^

Environmental cleaning methods that include cleaning, wiping, spraying, and use of additional technologies such as ultraviolet (UV) irradiation, aim to prevent the spread by contact with contaminated surfaces, regardless of the source. The World Health Organization (WHO) currently advises that for COVID-19, cleaning and disinfection in healthcare settings should be carried out in accordance with standard precautions, while community members are encouraged to adhere to routine practices. Coronavirus disease 2019 (COVID-19) virus is vulnerable to surfactants in cleaning agents and disinfectants.^4^

Despite the existence of internationally recommended guidelines on COVID-19 IPC,^4^ there are concerns regarding the adherence of health and care workers and the general population to these measures, particularly in relation to cleaning and disinfection. Mere knowledge of guidelines and interventions does not guarantee compliance. Understanding the experiences and perspectives of individuals can offer valuable insight into the facilitators and barriers related to adherence to cleaning and disinfection. Furthermore, these perceptions and factors influencing the uptake of these interventions may vary by facility, household and community due to several contextual factors.

An earlier qualitative systematic review on IPC guideline adherence, notably for respiratory infectious diseases, did not have any studies covering COVID-19 and focused only on healthcare setting studies.^5^ To address this gap, the review was commissioned by the WHO as part of the evidence-base for the 2023 update of the guideline on infection prevention and control in the context of COVID-19.^4^

When developing international guidelines, gaining a deeper understanding of individuals’ experiences, perspectives, beliefs, and priorities enhances the guidelines’ relatability, adaptability, and feasibility.^8^ Furthermore, studies have also shown even with international, national, and hospital policies on cleaning and disinfection, adherence to the intervention remains variable,^5,6,7^ and thus critical to provide context considerations to guideline panels.

The aim of this study is to appraise and synthesise the findings of qualitative studies on the perceptions of stakeholders and factors influencing the uptake of cleaning and disinfection in healthcare and community settings in the context of COVID-19.

## Methods

### Design

This rapid qualitative evidence synthesis (QES) was carried out within a limited time frame for synthesising evidence for updating the WHO guideline on IPC in the context of COVID-19.

Cochrane methods on conducting rapid QES were followed,^9,10^ and the reporting of the review was performed in line with the Preferred Reporting Items for Systematic Reviews and Meta-analyses (PRISMA) guidelines and the enhancing transparency in reporting the synthesis of qualitative research (ENTREQ) checklist.^11,12^ The QES protocol was registered on the International Prospective Register Of Systematic Reviews (PROSPERO) (CRD42022356474) prior to commencing the synthesis.

### Inclusion criteria

All primary studies that used qualitative methods for both data collection (e.g., focus group discussions, individual interviews) and for data analysis (e.g., thematic analysis, framework analysis) and written in English language only were considered. Mixed method studies whose qualitative components were collected and analysed using qualitative research methods were also considered. Therefore, studies that did not collect data through these qualitative methods were not included. Studies were not excluded based on the assessment of their methodological limitations.

The eligibility criteria were developed following the setting, perspective, phenomenon of interest, comparison and evaluation (SPICE) framework.^13^

#### Setting

Healthcare facilities including care homes; community and household settings.

#### Perspectives

Stakeholders including health and care workers irrespective of function (involved in patient care or not), healthcare policy makers, health facility clients and visitors, community members or general public, and members of households.

#### Intervention

Cleaning and disinfection interventions (cleaning, wiping, spraying, UV irradiation) for COVID-19 IPC.

#### Comparison

Not applicable for qualitative evidence synthesis.

#### Evaluation

Outcomes were evaluated broadly under two areas:

- Stakeholders’ perceptions including views, attitudes, perceptions, experiences and perspectives.
- Factors influencing uptake of the interventions were considered at different levels in line with the Supporting the Use of Research Evidence (SURE) framework.^14,15^ The SURE framework identifies factors that influence the implementation of a policy option at different levels such as the recipient of care, providers of care, other stakeholders, health system constraints, and the social and political constraints levels.

### Search methods

#### Information sources and search

Information scientists from Cochrane organisation identified keywords and synonyms relevant to the inclusion criteria and designed the search strategy based on these keywords (see Online Appendix 1). Two electronic databases: MEDLINE (Ovid) and WHO COVID research database were searched due to time constraints. Databases were searched from 01 January 2020 to 01 September 2022 (MEDLINE) and 02 September 2022 (WHO COVID research database). Furthermore, the reference lists of all the included studies and key references were reviewed to identify any additional primary studies for inclusion.

### Data collection and synthesis

#### Screening and selection of studies

After deduplication of the search output using Endnote reference management software,^16^ the eligibility criteria were applied to the remaining records. Title and abstract screening of the search output was carried out by four authors (A.N.M., E.C.O., M.A.O., H.N.O.) working in pairs, with one author screening all titles, abstracts, and full texts of potentially eligible studies using a pre-piloted eligibility screening form. The second author in each pair verified all screening output. Subsequently, studies that were considered relevant were included for the full text eligibility screening, which was carried out in pairs. At every stage, in case consensus was not reached between the two reviewers in including a study, a third reviewer (O.N.O.N.) made the inclusion or exclusion decision.

### Data extraction

A pre-piloted data extraction spreadsheet in Microsoft Excel was used to extract information on key study characteristics of the included studies. Two further spreadsheets were used to extract themes and supporting quotes relevant to the review objectives. One review author (H.N.O.) extracted data independently from included studies using a pre-piloted data extraction form. A second review author (A.N.M.) verified all extracted data for accuracy and completeness. Discrepancies and errors were discussed between the two reviewers for consensus or when required by further discussion with a third reviewer (O.N.O.N.).

### Assessment of methodological limitations of included studies

Two review authors (H.N.O., O.N.O.N.) independently assessed the methodological limitations of the included studies using an adapted version of the generic Critical Skills Appraisal Programme (CASP) qualitative studies checklist.^17^ This adapted checklist has been used in other qualitative evidence syntheses.^5^ Discrepancies in assessment were resolved through discussion with a third author (A.N.M.).

### Data analysis

For the synthesis of qualitative data, the thematic framework analysis approach was used to synthesise data drawing on the SURE framework.^18^ Using this approach we developed and organised findings into themes using inductive and constant comparison methods.^19^ The thematic synthesis approach is recommended by the Cochrane Qualitative Review Methods Group.^20^ The following steps were carried out for the synthesis: Firstly, one reviewer (O.N.O.N.) chose one of the article judged to most closely answer the review objectives and familiarised himself with the data. Secondly, this article was coded using a thematic analysis approach. Thirdly, the codes that emerged from step two were used to create a data extraction sheet while considering the SURE framework levels and domains. Fourthly, the next article was coded using the data extraction sheet. If necessary, additions were made to the data extraction sheet if new themes emerged from the subsequent articles. Finally, the process was continued until data was extracted from all of the sampled articles. Three other authors verified data extraction and added any other data that they felt should have been included. Themes from the coded data identified were aggregated and critically examined to develop analytical themes for the review findings.

### Assessing confidence in the review findings

The *Grading of Recommendations, Assessment, Development, and Evaluations –* Confidence in the Evidence from Reviews of Qualitative Research (GRADE-CERQual) approach^21^ was used to assess the confidence level (high, moderate, low or very low) in each review finding. This assessment was made across four domains (methodological limitations of included studies, coherence of the review finding, adequacy of the data contributing to a review finding, and relevance of the included studies to the review question). At least two review authors assessed the confidence of each finding across the four domains. The overall assessment was based on the consensus of the author team.

### Review authors’ reflexivity

The author team is a multidisciplinary team of researchers, some with healthcare background, others with social science background, and some with experience in conducting qualitative research, synthesis of effectiveness studies, and epidemiology. Most of the review authors also have considerable knowledge of existing social behavioural conceptual frameworks that explain health behaviour. Throughout the data synthesis, the authors especially those working in the health field were aware of their own positions and reflected on how these could influence the data synthesis and study design. With an aim of identifying assumptions in the data synthesis, we also presented the preliminary findings to experts on COVID-19. We expect our collective and individual experiences of the pandemic and interest (as researchers, clinicians, and academics) have enabled us to provide rich insights and balanced views with respect to the conduct of the review, consideration of the findings and interpretation of the evidence.

## Results

**FIGURE 1:**
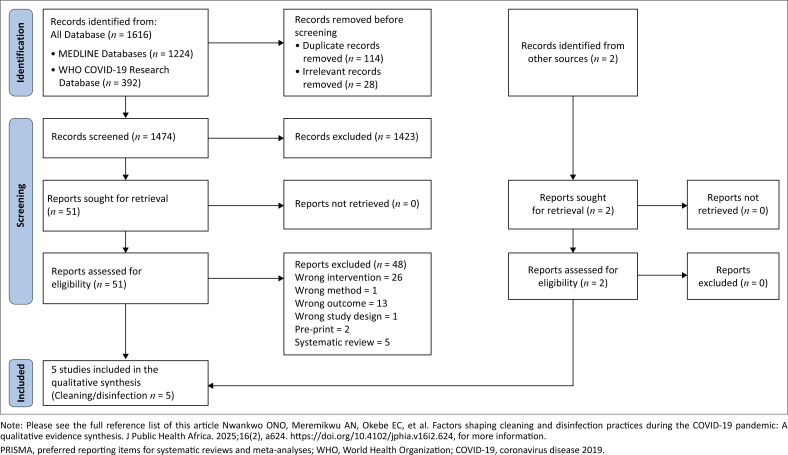
Preferred reporting items for systematic reviews and meta-analyses flow diagram.

The search retrieved 1618 studies from the databases and a secondary reference search. Of which, 53 studies were retrieved for full screening whereas 48 were excluded (Online Appendix 2). We included five studies in our final review as shown in the PRISMA flow diagram (see Figure 1).

All five studies that met our inclusion criteria provided insights into cleaning and disinfection interventions.^22,23,24,25,26^ There were no studies found addressing the issues of spraying, UV irradiation nor on differential/additional cleaning surfaces interventions. The studies were published between 2021 and 2022. Two studies were carried out in lower-middle income countries (LMICs): Ethiopia^23^ and Ghana,^22^ and three in high-income countries (HICs): Australia,^24^ Denmark^25^ and the United Kingdom.^26^ Three studies were carried out in health facilities^22,23,24^ while two studies were in community or household settings.^25,26^ Furthermore, three studies included the perspectives of health and care workers,^22,23,24^ one included the perspectives of short-term accommodation owners (Air BnB [bed and breakfast] hosts)^25^ and the primary school head teachers^26^ (see Table 1).

**TABLE 1:** Characteristics of included studies (*n* = 5).

Author (year); country	Setting	Participants	Perspective	Data collection method	Key outcomes
**Cleaning and disinfecting (*n* = 5)**					
Afeng-Nkansah (2021)^22^; (Ghana)	Facility (military hospital)	Nurses/emergency medical technicians in triage unit, and clinicians/nurses/intensive care staff in holding unit and treatment centre (*n* = 41 observations)	Health workers	Observations	Health worker response and adherence to COVID-19 protocols
Amera Birlie (2021)^23^; (Ethiopia)	Facility (specialised hospital)	Staff nurses (*n* = 76 observations); head nurses (*n* = 6 interviews) in inpatient and outpatient services	Health workers	Observations; interviews	Nurses’ cleaning practice of non-critical medical equipment
Curryer (2021)^24^; (Australia)	Facility	Registered and enrolled nurses and midwives (*n* = 6)	Health workers	Interviews	Knowledge and experiences of infection prevention and control processes and cleaning Perceptions about workplace risk-management during COVID-19
Fischer (2022)^25^; (Denmark)	Community	Air BnB hosts (*n* = 10)	General public	Interviews	Experiences with hosting on the Airbnb platform, and adaptation of hospitality practices during the pandemic
Sundaram (2021)^26^; (United Kingdom)	Community	School headteachers (*n* = 14)	Community	Interviews	Feasibility of implementing preventive measures to prevent SARS-CoV-2 transmission

Note: Please see the full reference list of this article Nwankwo ONO, Meremikwu AN, Okebe EC, et al. Factors shaping cleaning and disinfection practices during the COVID-19 pandemic: A qualitative evidence synthesis. J Public Health Africa. 2025;16(2), a624. https://doi.org/10.4102/jphia.v16i2.624, for more information.

COVID-19, coronavirus disease 2019; SARS-CoV-2, severe acute respiratory syndrome coronavirus 2; BnB, bed and breakfast.

The methodological quality of the studies ranged from minor methodological limitations (4 studies)^22,23,24,26^ to moderate limitations (1 study).^25^ Most studies provided descriptive information on the study context, sampling strategy, data collection and analysis approaches, and ethical considerations. They also offered underlying data to support their findings. None of the studies explicitly reported on the author’s reflexivity (see Table 2).

**TABLE 2:** Methodological quality of included studies.

Author(s), year	Was the setting(s) and context adequately described	Was the sampling strategy appropriate and described?	Was the data collection strategy appropriate and described?	Was the data analysis appropriate and described?	Were the findings (claims made) supported by evidence?	Is there evidence of researcher reflexivity?	Have ethical issues been taken into consideration?	Any other concerns	Overall assessment of methodological limitations
Afeng-Nkansah (2021)^22^	Yes	Yes	Yes	Yes	Yes	No	Yes	No	Minor
Amera Birlie (2021)^23^	Yes	Yes	Yes	Yes	Yes	No	Yes	No	Minor
Curryer (2021)^24^	Yes	Yes	Yes	Yes	Yes	No	Yes	No	Minor
Fischer (2022)^25^	Yes	Yes	Yes	Yes	No	No	Yes	No	Moderate
Sundaram (2021)^26^	Yes	Yes	Yes	Yes	Yes	No	Yes	No	Minor

Note: Please see the full reference list of this article Nwankwo ONO, Meremikwu AN, Okebe EC, et al. Factors shaping cleaning and disinfection practices during the COVID-19 pandemic: A qualitative evidence synthesis. J Public Health Africa. 2025;16(2), a624. https://doi.org/10.4102/jphia.v16i2.624, for more information.

Based on our assessment on the level of confidence in the review findings using the GRADE-CERQUAL approach,^20^ five findings were rated as low confidence and one as moderate confidence. The key concerns were data adequacy and relevance.

The summaries of the findings and assessments of confidence in these findings are presented in the Summary of Qualitative Findings (SoQF) table (see Table 3).

**TABLE 3:** A summary of qualitative findings.

S/N	Summary of review finding (analytical theme)	Studies contributing to this review finding	CERQual assessment	Explanation of CERQual assessment
1	Cleaning is a cornerstone of patient care	Curryer et al.^24^ (Australia)	Low confidence	Minor concerns regarding methodological limitations and coherence, moderate concerns regarding adequacy and relevance (one qualitative study contributing thin data to this finding and limited geographical spread)
2	Organisational culture affects cleaning practices	Curryer et al.^24^ (Australia)	Low confidence	Minor concerns regarding methodological limitations, moderate concerns regarding coherence (some concern about the fit between the data from primary studies and the review findings) relevance and adequacy (one qualitative study contributing thin data to this finding and the other qualitative study providing rich data)
3	Individual judgement influences uptake	Afeng-Nkansah et al.^22^ (Ghana)	Low confidence	Minor concerns regarding methodological limitations and coherence, moderate concerns regarding adequacy and relevance (one study contributing thin data to this finding and limited geographical spread)
4	Historic standards, norms and practices influence uptake	Curryer et al.^24^ (Australia)	Low confidence	Minor concerns regarding methodological limitations and coherence, moderate concerns regarding adequacy and relevance (one qualitative study contributing thick data to this finding and limited geographical spread)
5	Pivoting practice quickly as guidance changed	Curryer et al.^24^ (Australia); Fischer and Roelofsen^25^ (Denmark);	Low confidence	Minor concerns regarding methodological limitations, coherence and moderate concerns regarding adequacy and relevance (only 2 studies from HIC countries)
6	Resource considerations for cleaning uptake	Amera Birlie et al.^23^ (Ethiopia); Curryer et al.^24^ (Australia); Sundaram (2021)^26^ (United Kingdom)	Moderate confidence	Minor concerns regarding methodological limitations and coherence. Moderate concerns regarding adequacy and relevance (3 studies with two from HIC and 1 from LMIC providing moderately rich data)

Note: Please see the full reference list of this article Nwankwo ONO, Meremikwu AN, Okebe EC, et al. Factors shaping cleaning and disinfection practices during the COVID-19 pandemic: A qualitative evidence synthesis. J Public Health Africa. 2025;16(2), a624. https://doi.org/10.4102/jphia.v16i2.624, for more information.

S/N, serial number; CERQual, confidence in the evidence from reviews of qualitative research; UK, United Kingdom; HIC, higher income countries; LMICs, lower-middle income countries.

### Qualitative synthesis findings

Six findings (analytical themes) were identified, focusing on cleaning and disinfection interventions. The analytical themes and corresponding descriptive themes are presented with their sample quotes in Table 4. The findings are as follows:

**TABLE 4:** Synthesis results (themes and supporting quotes).

S/N	Analytical themes	Descriptive themes (review findings)	Studies contributing to the review finding	Supporting data (example quote)
**Cleaning and disinfection**				
1	Cleaning is a cornerstone of patient care	Importance of cleaning recognised (health workers)	Curryer et al.^24^ (Australia)	‘Our duty of care to our patients is to ensure that they’ve got the best environment … it’s all about preventing, doing what you can to prevent any infection to occur to the patient …’ (Curryer, 2021, Australia)
2	Organisational culture affects cleaning practices	Cleaning practices are undervalued (by management) (health workers)	Curryer et al.^24^ (Australia)	‘Getting management to understand why I might need [*time for cleaning*] … that’s a challenge when they’re non-clinical; [*Cleaning is*] highly underrated and extremely vital.’ (Curryer, 2021, Australia)
3	Individual judgement influences uptake	Differential disinfection practices in risk areas of facility (health workers)	Afeng-Nkansah et al.^22^ (Ghana)	Regular disinfection of surfaces at the wards of the treatment centre was observed; however, the restrooms and changing rooms and working surfaces were not disinfected frequently (Afeng-Nkansah, 2021, Ghana)
4	Historic standards, norms and practices influence uptake	COVID-19 exposed/cracked open poor cleaning practices and standards (health workers)	Curryer et al.^24^ (Australia)	‘It seemed that COVID-19 had finally ‘cracked open’, brought to light the “rot” and “poor infection control practices [*already*] in existence” (P3) within the healthcare system: ‘You look at aged care, you look at EDs, our patient toilets have always been dirty, our staff toilets are dirty, our staff tea rooms are dirty. All COVID’s done is just cracked it wide open.’ (Curryer, 2021, Australia)
5	Pivoting practice quickly as guidance changed	A lack of clear guidance/changing guidance (community/health workers)	Curryer et al.^24^ (Australia)Fischer (2022)^25^ (Denmark)	Nurses described having to quickly pivot practices in response to emerging evidence about COVID-19, changes in recommended guidelines and working conditions, and disrupted supplies. Lacking a firm foundation from which to practice, nurses were left negotiating shifting sands: ‘one day we are being told to do one thing and then the next day do something else.’ (Curryer, 2021, Australia)
6	Resource considerations for cleaning uptake	Resources required for disinfecting and cleaning (health workers/community)	Amera Birlie et al.^23^ (Ethiopia); Curryer et al.^24^ (Australia); Sundaram (2021)^26^ (UK)	Economic priorities meant that less than 3 min cleaning and preparation time was allowed between patients: ‘they’re all time-based appointments. So it’s trying to figure out how [*cleaning*] can be done and not upset the [*patient*] booking’ (Curryer, 2021, Australia) In several schools, a lack of budget meant that teachers, teaching assistants and school leaders had to do the cleaning themselves (Sundaram, 2021, UK)

Note: Please see the full reference list of this article Nwankwo ONO, Meremikwu AN, Okebe EC, et al. Factors shaping cleaning and disinfection practices during the COVID-19 pandemic: A qualitative evidence synthesis. J Public Health Africa. 2025;16(2), a624. https://doi.org/10.4102/jphia.v16i2.624, for more information.

S/N, serial number; COVID-19, coronavirus disease 2019; UK, United Kingdom; EDs, emergency departments.

#### Finding 1: Cleaning is a cornerstone of patient care (low confidence)

Health and care workers in Australia recognised the importance of cleaning as a bedrock of patient care and prevention of disease.^24^ One health and care worker states, ‘Our duty of care to our patients is to ensure that they’ve got the best environment … it’s all about preventing, doing what you can to prevent any infection to occur to the patient …’.^24^

#### Finding 2: Organisational culture affects cleaning practices (low confidence)

One study, done in Australia, reflected on how the actions or inactions of management can influence the approach health and care workers may take in regard to cleaning practices. The participant noticed that challenges with management, who undervalued the importance of cleaning, affected individual health and care workers’ performance.^24^ They note, ‘Getting management to understand why I might need [*time for cleaning*] … that’s a challenge when they’re non-clinical; [*Cleaning is*] highly underrated and extremely vital’.^24^

#### Finding 3: Individual judgement influences uptake (low confidence)

Despite the value placed upon cleaning and disinfection, one cannot discount the impact of individual judgement on the adoption of cleaning and disinfection practices. One study reported variation in cleaning and disinfection practices among health and care workers according to individual perceived risk.^22^ The study observed a higher compliance to disinfection and cleaning protocols in areas of the health facilities with a higher risk of infection (e.g., treatment centre for COVID-19) compared to the lower infection risk areas (e.g., triaging area).^22^

#### Finding 4: Historic standards, norms and practices influence uptake (low confidence)

The emergence of COVID-19 uncovered longstanding inadequacies in cleaning practices within healthcare facilities, highlighting systemic deficiencies in cleaning and disinfection protocols. A study from Australia described how COVID-19 amplified the existing shortcomings in cleaning and disinfection practices within the health system.^24^ The study points to the fact that although COVID-19 may have raised awareness about IPC, it may have not been able to overcome the historical lack of hygiene practices in the institutions. This is illustrated by this quote from a health worker:

It seemed that COVID-19 had finally ‘cracked open’, brought to light the ‘rot’ and ‘poor infection control practices [*already*] in existence’ within the healthcare system: ‘You look at aged care, you look at EDs [*emergency departments*], our patient toilets have always been dirty, our staff toilets are dirty, our staff tea rooms are dirty. All COVID’s done is just cracked it wide open.^24^

#### Finding 5: Pivoting practice quickly as guidance changed (low confidence)

Two studies one from the healthcare area^24^ and the other from the community^25^ illustrated the importance of having clear guidance on cleaning and disinfection during the pandemic. The evolving guidelines created a challenge for health and care workers, as they needed to change practices to align with the latest recommendation. Nurses and midwives in Australia described frustration with this situation, noting the difficulties faced with continuously adjusting their practices based on emerging evidence and update guidelines on COVID-19.^24^ One of them aptly described the situations thus ‘[COVID-19] is an evolving issue … one day we are being told to do one thing and then the next day do something else’.^24^

#### Finding 6: Resource considerations for cleaning uptake (moderate confidence)

Three studies pointed out how resources availability for implementing cleaning and disinfection was a major consideration in both health facilities as well as other public places such as schools.^23,24,26^ This consideration limited the scope of cleaning and disinfection carried out as well as made some reallocation of resources necessary in order to ensure proper cleaning. The study performed in the United Kingdom noted, ‘In several schools, a lack of budget meant that teachers, teaching assistants and school leaders had to do the cleaning themselves’.^26^

## Discussion

We have carried out a QES on factors influencing the uptake of cleaning and disinfection interventions in the context of the COVID-19 pandemic. No qualitative studies were found in this review for spraying, wiping and spraying, UV irradiation and differential cleaning.

The findings highlight that cleaning is regarded as a cornerstone of patient care, with organisational culture influencing cleaning practices. A barrier identified is the substantial resource demands associated with cleaning and disinfection processes. Notably, the findings primarily reflect the perspectives of health and care workers, offering limited insights from the community.

Consistent with the present findings, a previous review examining other respiratory infectious diseases, excluding COVID-19, highlighted the challenges health and care workers faced in implementing IPC guidelines, particularly the rapid pace at which some guidelines evolved.^5^ This corresponds to the current finding that the rapid change in guidelines during the COVID-19 pandemic, affected the clarity of guidance for health and care workers, thereby impacting on their understanding of proper cleaning and disinfection procedures. These findings also suggest that emphasis should be placed at all levels – national, subnational, and healthcare facilities – to ensure clear and concise communication about policy changes, along with any required additional training. Furthermore, additional findings remain consistent with previous reviews, showing that hospital management attitude to cleaning and disinfection can affect health and care worker’s uptake and implementation of cleaning.^5,27^

One pivotal finding from this work is the need for adequate resources to be available for encouraging uptake of cleaning and disinfection. This supports earlier findings from other reviews, which highlight the critical role resource issues constraints play in the adoption and implementation of interventions.^5,27^ Furthermore, a narrative synthesis by Palagyi and colleagues^28^ similarly identified considerable organisational, environmental and individual factors that can affect health and care workers’ ability to comply with guidelines. However, their study was looking at health system preparedness for emerging infectious diseases in low- and middle-income countries.

No direct qualitative evidence was found on stakeholders’ perceptions and experiences or contextual factors influencing the uptake of spraying, UV irradiation and disinfection by using differential/additional cleaning of surfaces. This may not be unexpected as these are more structural/mechanical interventions that are less likely to require end-user behaviour change.

### Implications for practice

This review highlights important considerations for implementing IPC interventions in the context of COVID-19. A key finding indicates that resource availability, particularly human resources, affects the implementation and compliance with cleaning and disinfection guidelines. Therefore, implementers and policymakers should anticipate the significant resource needs and make arrangements that can be mobilised on very short notice. This underscores the importance of long-term contingency planning to ensure resources are readily accessible when outbreaks such as COVID-19 arise. Another notable finding is the need to address pre-existing structural and organisational barriers to IPC implementation in facilities. Shifting institutional norms and fostering an organisational culture that values patient safety and IPC practices may further support adherence to best practices.

### Limitations of the study

This QES was carried out as a rapid review and hence some compromises were made in order to provide the evidence in a timely manner such as searching only two databases, limiting the studies to English articles and randomly double-screening titles and abstracts. This may have limited potential studies that may have otherwise been included; however, a secondary reference search of the included studies and other related systematic reviews was completed for thoroughness. Again, there were very few studies contributing to each of the review findings, which may not be surprising given the subject matter. Notwithstanding these limitations, the review team maintained methodological rigour in conducting the review by adhering to standard Cochrane Methods,^9^ which reduced the bias that may be associated with studies of this nature.

## Conclusion

This review has identified several factors influencing the uptake of cleaning and disinfection interventions in the context of the COVID-19 pandemic, although the number of studies was limited. Resource requirements emerged as a significant finding that can impact the implementation of cleaning and disinfection practices both in the community and healthcare facilities. This critical insight should be considered by policymakers and healthcare administrators when developing policies and programmes for cleaning and disinfection. Initiatives that ensure adequate resources, including human resources and supplies, may be more effective in enhancing cleaning and disinfection practices.

Given the limited availability of data, further qualitative studies are necessary to explore the barriers and facilitators related to cleaning and disinfection. These studies are vital to creating a well-documented and comprehensive perspective that can guide strategies to improve adherence to policies by pinpointing specific challenges and effective practices that encourage compliance. Additionally, more studies that include an equity perspective, are needed, as the predominant majority of the studies did not consider the role of gender, socioeconomic status and social determinants among others on the outcome. Most of the included studies were conducted in HICs. This research should also address how interventions such as wiping and spraying, UV irradiation, and differential cleaning are being implemented.
